# Zur Rolle von Unterhaltungsmagazinen in der Pandemie. Zwischen entdifferenzierten Sammelsurien und differenzierten Wissensvermittlern

**DOI:** 10.1007/s41244-022-00260-4

**Published:** 2022-07-19

**Authors:** Maren Lickhardt

**Affiliations:** Institut für Germanistik, Leopold-Franzens-Universität Innsbruck, Innsbruck, Österreich

**Keywords:** Unterhaltungszeitschriften, Covid-19, Wissenschaftsvermittlung, Kontaktszenen, Boulevardmedien, Popular Press, Entertainment Press, Covid-19, Science Communication, Contact Scene

## Abstract

Dieser Artikel untersucht, wie illustrierte Unterhaltungsmagazine Covid-19 in zwei Zeiträumen thematisieren: von Februar bis Juli 2020 und von Oktober bis Dezember 2021. Die Zeitschriften stellen nicht nur eine Kontaktszene zwischen Wissenschaft und Öffentlichkeit dar, sondern vermitteln auch zwischen verschiedenen professionellen Diskursen, die die Pandemie bearbeiten, und müssen selektieren, welchen Bearbeitungsmodus sie präferieren. Während sich die Zeitschriften, weil sie sich ohnehin im permanenten Krisenmodus befinden, zunächst insofern kaum von der Pandemie irritieren lassen, als sie diese im boulevardesken Modus bearbeiten, indem sie etwa die Reaktion von Prominenten zeigen, die ihrerseits Laien sind, stellen sie in der zweiten Untersuchungsphase auf seriöse Vermittlung von Wissenschaft um und tragen zur Popularisierung von medizinischem und epidemiologischem Wissen bei.

## Abstract (en)

This article explores how illustrated entertainment magazines address Covid-19 in two time periods: from February to July 2020 and from October to December 2021. The journals not only represent a contact scene between science and the public, but also mediate between various professional discourses that deal with the pandemic and have to select which processing mode they prefer. While the journals, because they are already in permanent crisis mode, are initially hardly irritated by the pandemic in that they work on it in tabloid mode, for example by showing the reaction of celebrities who are themselves laymen, in the second phase of the investigation they switch to serious communication of science and contribute to the popularization of medical and epidemiological knowledge.

## Vorbemerkungen

Die öffentliche Diskussion um Covid-19 dreht sich in großem Maß um die Frage nach Kompetenz und Zuständigkeit, weil eine Pandemie zu komplex ist, um nur von einem gesellschaftlichen System bearbeitet werden zu können (vgl. Werber/Lickhardt 2010): Medizin, Naturwissenschaft, Politik, Ökonomie und viele mehr sind in dem Zusammenhang gefragt. Jedes System hat sein eigenes Programm, um auf die Pandemie zu reagieren. Aber abgesehen davon, dass jedes einzelne zu unterkomplex bzw. begrenzt ist für die alle gesellschaftlichen Bereiche durchdringende Pandemie, muss ausgehandelt werden bzw. wurde in allen Phasen der Pandemie ausgehandelt, wer wie viel zu sagen haben sollte, welchem Bereich Priorität eingeräumt wird. Während die einen den Geltungsanspruch virologischer und epidemiologischer Erkenntnisse nicht anzweifelten und als Maßstab politischen, gesamtgesellschaftlichen und individuellen Handelns erachteten, brachten andere wirkliche oder vermeintliche ökonomische Erfordernisse in Anschlag. Die Diskussion um die Pandemie ist ein gutes Beispiel dafür, wie diese systemtheoretisch inspirierten Erwägungen im beobachteten Feld selbst vorgefunden werden können, ohne dass alle Diskutierenden diese kennen, weil hier explizit mit dem Wissen darum, dass die Operationen des einen Systems mit denen eines anderen oftmals unvereinbar sind, um deren Geltungsbereiche gestritten wurde. Noch viel heftiger als die Geltungsbereiche einzelner Systeme wurde die Frage debattiert, wie und mit welchem Recht die Pandemie im Populären ausgetragen wird. Hierbei ging es besonders um unzuständige Dilettant:innen aller Art, die auf diversen Kanälen mit Pseudo-Wissen und Lai:innen-Empfindungen aufwarteten, um schädliche Heilslehren zu verbreiten und rechtspopulistische Verschwörungsnarrative zu normalisieren. Nicht selten war die Rede von der »YouTube Universität« (Heilrath 2022) oder »Telegram-Universität« (Typ 2022), wobei selbstverständlich kein Medium per se nützlich oder schädlich ist und auch seriöse Akteur:innen und Institutionen soziale Medien nutzen. Insgesamt hat die Pandemie die diskursiven und systemischen Rollenverteilungen erschüttert, und es war und ist irritierend zu beobachten, dass zumindest wir in Europa oder überhaupt in den so genannten westlich zivilisierten Gesellschaften diese Diskussionen statt eines Skriptes zur Bewältigung der Situation hatten und haben. Über Vertrauensverluste und Politikverdrossenheit ist im Kontext der Pandemie häufig gesprochen worden, und vielleicht resultieren diese Gemütslagen weniger aus einzelnen Entscheidungen, die jeweils den einen passen und den anderen nicht, als vielmehr aus den systemischen Erschütterungen angesichts dieses übersystemischen Phänomens. Die Grenzen der Systeme und ihre jeweils schlechte Anschlussfähigkeit wurden sichtbar, weil es besagtes Skript nicht gab. Aber hierin liegt auch eine Chance, weil grenzüberschreitende Kontaktaufnahmen zu einer Refiguration aller beteiligten Systeme führen können.

Covid ist ein Phänomen, das in spezialisierten Wissenschaften beobachtet, von der Politik gehandhabt und letztlich im Populären ausgetragen werden muss. Nicht nur wollte die Öffentlichkeit von Beginn an informiert werden, sondern sie musste auch mit ihrem Verhalten zum – zumindest anfangs noch erhofften – Gelingen der Pandemieeindämmungsmaßnahmen beitragen. Aufgrund der Notwendigkeit beider Kommunikationsrichtungen sahen sich spezialisierte Expert:innen entgegen ihrer Gewohnheit gezwungen, an den populären Grenzen ihrer Disziplinen und darüber hinaus zu operieren, so zum Beispiel Christian Drosten und Sandra Ciesek. Mit ihrem vom NDR kuratierten Podcast waren sie nicht nur zwei der ›Expert:innen für Pandemie‹ schlechthin, sondern sie wurden zu direkten Medialisierer:innen und Popularisierer:innen (vgl. Venus 2022). Zwischen der besagten Kommunikation in unseriösen Messenger-Dienst-Kanälen und dieser direkten Wissenschaftsvermittlung spielten und spielen aber nach wie vor traditionelle massenmediale Gatekeeper eine Schlüsselrolle, und dabei muss beachtet werden, dass sie selbstverständlich einen komplexeren Zugang zur Pandemie oder überhaupt eine andere Funktion haben, als Wissenschaftsvermittlung zu betreiben. Das bedeutet, dass sich traditionelle Massenmedien – vermutlich eher als einzelne Accounts in sozialen Medien – *eo ipso* als Kontaktfläche anbieten, um verschiedene systemische Anforderungen mindestens additiv in verschiedenen Ressorts und Berichten, aber teilweise auch in synthetisierenden Reflexionen in einzelnen Artikeln zusammen zu bringen.

Massenmedien bilden einen eigenständigen, mit Politik, Wissenschaft und Wirtschaft idealerweise nur indirekt verbundenen gesellschaftlichen Apparat mit spezifischen Funktionen: Sie sortieren Ereignisse nach Nachrichten- und Unterhaltungswert und bestimmen die gesellschaftliche Agenda mit; sie dürfen Ereignisse für die von ihnen hergestellte Öffentlichkeit kommentieren; und sie verleihen dieser Öffentlichkeit über diese Kommentierungen eine mehr oder weniger authentische Stimme. Massenmedien entscheiden selbständig, was sie popularisieren,^1^ und haben daher keinen geringen Stellenwert, wenn es um die Frage geht, was allgemein, im Populären über Covid kursiert. Boulevard-Printmedien wie Mode‑, Lifestyle- sowie vor allem Klatsch und Tratsch-Zeitschriften – ein weites Spektrum, das noch zu differenzieren sein wird – sind ihrerseits je heterogene Gebilde, aber sie haben eine einheitliche Funktion: Sie operieren im Populären oder generieren das Populäre; sie verbreiten, verstärken oder erschaffen, was populär ist in dem Sinne, was von vielen beachtet wird (vgl. Hecken 2006, S. 85; Döring/Werber/Albrecht-Birkner et al. 2021, S. 4). Dabei stellen sie eine Kippfigur zwischen Spezialisierung und Entdifferenzierung dar: Sie dienen weniger als andere Massenmedien der Information, weil es ihnen nicht um das Neue geht, sondern um die Sensationalisierung und Spektakularisierung des Bekannten; sie dienen der Unterhaltung in dem Sinne, dass sie auf eine reizvolle Weise für möglichst viele möglichst anschlussfähig sein, also den Common Sense bestätigen müssen. Alles kann bzw. insbesondere alle alltagsbezogenen Lifestyle-Fragen können Gegenstand von Boulevardmedien sein, sodass sie ein entdifferenziertes Tableau an Themen liefern können, ohne dabei auf ihre spezifische Funktion verzichten zu müssen, auf unterhaltende Weise Konsonanz zu erzeugen oder auf konsonante Weise zu unterhalten. Das heißt, sie leben von dem Paradoxon, Erwartbares überraschend zu gestalten, wozu sie aus dem Populären aller Systeme schöpfen und dabei stets mehr oder weniger im Krisenmodus agieren. Strukturell davon unterschieden ist die Tatsache, dass sie aufgrund ihrer mittleren Frequenz – sie erscheinen regelmäßig, aber nicht täglich – ebenfalls paradoxerweise Aktualität kommunizieren, obwohl sie nicht immer, aber oft Zweitverwertungen liefern. Darüber hinaus haben Zeitschriften eine Orientierung stiftende und Rat gebende Funktion. Dies ergibt sich nicht nur über je spezialisierte Ressorts, zum Beispiel Gesundheitsratgeber, sondern auch über Werbung und sämtliche redaktionelle Teile, in denen Lifestyles vorgelebt werden. Außer in speziellen Ressorts wie den genannten Gesundheitsratgebern geht es prinzipiell in Unterhaltungszeitschriften weniger um Expert:inneneinschätzungen als um Rollenvorbilder aus dem Kreis der Laien, mit denen sich Leser:innen identifizieren können. So ist die *Freundin* schon qua Titel die Freundin der Rezipient:innen, *Brigitte* und *tina* sogar namentlich gekennzeichnet. Diese populären Medien können keinen Expertenstatus in Bezug auf die meisten Themen und schon gar nicht auf Covid für sich beanspruchen, auch wenn in einzelnen Ressorts Expert:innen zu Wort kommen können, und sie sind auch nicht dafür zuständig, Covid überhaupt zu thematisieren, kommen aber letztlich gerade aufgrund ihrer mangelnden Spezialisierung und ihrem mittleren Aktualitätsgrad nicht um dieses Phänomen herum.

In Bezug auf die Inkommensurabilität der Anforderungen verschiedener gesellschaftlicher Systeme bietet sich die Bildlichkeit der Systemtheorie an. In Bezug auf Massen- insbesondere Boulevardmedien eignet sich die Vorstellung der Kontaktszene, wie sie im vorliegenden Heft verwendet wird, besser, weil Zeitschriften eine kontaktstiftende Schnittstelle zwischen verschiedenen Spezialdiskursen und zwischen Spezialdiskursen und dem Populären bilden. Gleichzeitig sind sie das Populäre selbst oder personifizieren es, auch indem sie reziproke Kommunikation auf zweierlei Weise betreiben: erstens in Form des Abdruckens von Leser:innenbriefe etc., zweitens indem implizite Leser:innen immer schon mitgedacht sind und dem Volk ›aufs Maul geschaut‹ wird. In gewisser Weise waren Zeitschriften schon vor den sozialen Medien ein Prosumer-Phänomen. Insofern diese Medien einerseits durchaus Belehrungen vornehmen können, sich aber andererseits in die Wünsche und Interessen der Rezipient:innen fügen, passen sie zu der Covid-Situation, dass *top down* Informationen im Dienste der Verhaltensmodifikation zu vermitteln waren, und *bottom up* ein Bedürfnis nach Information und Ratschlägen vorlag sowie Ventile für Pandemie-induzierte Befindlichkeiten gebraucht wurden. Aufgrund des breiten Leistungsspektrums dieser Medien ist zu erwarten, dass sie dynamisch auf die Situation reagieren, was im Begriff der Kontaktszene enthalten ist; während Systeme nur bedingt zu Kontaktaufnahmen im Sinne einer gleichberechtigten Kooperation fähig sind, dürfte es Zeitschriften per se nicht schwer fallen, sich auf multiple Interaktionen verschiedener Systemanforderungen einzustellen. Es wird daher im Folgenden von vornherein nicht um Wissen(schaft)svermittlung im Kontext von Covid, sondern um dessen Thematisierung gehen. Dabei wird zu fragen sein, wie groß der Anteil der Wissen(schaft)svermittlung ist und ob dieser im asymmetrischen Expert:innenmodus oder im symmetrischen ›Freund:innenschaftsmodus‹ vollzogen wird.

Mit diesen Überlegungen wurden zwischen Februar und Juli 2020 sowie Oktober bis Dezember 2021 zahlreiche, willkürlich ausgewählte Unterhaltungsmagazine gelesen, unter diesen jedoch keine für Spezialinteressen außer Mode. Insgesamt wurden ca. 120 Ausgaben durchgesehen, darunter in unregelmäßigen Intervallen: *Bild der Frau, Brigitte, Bunte, Closer, Cosmopolitan, Das Goldene Blatt, Das Neue Blatt, die aktuelle, Elle, Frau aktuell, Frau im Spiegel, Freizeit Revue, Freundin, Für Sie, Gala, Gala Style, Glamour, inTouch, Inside, In. Star News, InStyle, Jolie, MySelf, Neue Post, Neue Welt, neue woche, tina, Vogue, What’s up, Woche direkt*. Jede Erwähnung der Pandemie wurde, sofern es dem menschlichen Auge möglich ist, registriert und kategorisiert. Man kann von einer Mischung aus quantitativer und qualitativer Inhaltsanalyse sprechen, weil am Ende nicht exakt gezählt, sondern grob nach Häufigkeiten sortiert und nach eigenem Ermessen interpretiert wurde. Insofern liegt zwar eine quantitativ-empirische Datenbasis vor, die Auswertungen resultieren aber aus einer qualitativ-hermeneutischen Vorgehensweise.

## Phase Februar bis Juli 2020

Nicht wenige illustrierte Unterhaltungsmagazine thematisieren Covid in den ersten Wochen und Monaten auf dem Cover. Hier trifft die Krise nicht nur auf Prominente, sondern vor allem auf andere Krisen. Z.B. ist die ältere Generation der englischen Royals Covid-bedingt außer Gefecht gesetzt, und vor diesem Hintergrund kann umso mehr herausgestellt werden, wie pflichtbewusst William und Kate ihren royalen Pflichten nachkommen. Eigentlich geht es dabei aber um eine andere Krise, weil besonders auffallen kann, dass sich Harry und Meghan vom Königshaus abgewendet haben und entsprechenden Pflichten nicht nachkommen. Insgesamt werden in nahezu allen Magazinen nun die Beziehungen diverser Stars hinsichtlich der Frage durchdacht, wer angesichts der häuslichen Isolation nun zusammenbleibt und wer sich trennt. Aber weil Boulevardmedien das immer tun, ändert sich nicht viel, sondern es wird nun die Prominenz zusätzlich mit dem Populären der Pandemie aufgeladen. Dabei geht es weder um die Pandemie selbst noch bedürfen Prominente der Pandemie als besondere Würze. Es kommt vielmehr phatisch und metakommunikativ zum Ausdruck, dass man sich seitens der Redaktionen dessen bewusst ist, was ganz oben auf der Agenda steht, an was man also nicht vorbei kommt. Auch mit zahllosen Formulierungen wie »Auch in Corona-Zeiten […]«^2^ oder »Gerade in Zeiten wie diesen…«^3^, in denen es dann um Kochrezepte, Gymnastiktipps etc. geht, bestätigen die Zeitschriften den Leser:innen, dass man an der Zeit dran ist und Covid nicht vergessen hat, auch wenn die Selektionskriterien und die Themen zunächst einmal im Wesentlichen die gleichen geblieben sind. Das heißt, die Pandemie hat in der ersten Zeit trotz ihrer häufigen Thematisierung eine vergleichsweise geringe disruptive Kraft, weil die Magazine ohnehin im permanenten Krisenmodus operieren und sich die Pandemie additiv auf die üblichen Themen aufsetzen lässt. Die ratgebende Funktion der Zeitschriften hinsichtlich Gesundheitsfragen im spezialisierten Ressort Gesundheitsratgeber kommt in dieser Phase der Pandemie nicht zum Zug, weil die niedrige Frequenz der Zeitschriften dem äußerst dynamischen Geschehen nicht gerecht werden kann und zu diesem Zeitpunkt noch kaum wissenschaftliche Erkenntnisse oder politische Maßnahmen vorliegen, die nicht innerhalb von Tagen oder gar Stunden einer Revision unterzogen werden müssten. Insofern bleibt man bei der eigenen ›Spezialisierung‹ auf Lifestyle, Mode und Prominente. Es ist seriös, zu dieser Zeit das eigene Programm beizubehalten und im Gesundheitsressort weiterhin auf Rheuma- und Blasenbeschwerden zu setzen. Aber die Kombination von Pandemie und Populärem/Prominenz hat problematische Nebeneffekte.

Weil die Magazine ›Experten‹ für Beziehungsfragen sind und zu diesem Komplex routiniert Rat geben können, wird der Fokus auf die Frage gelenkt, wie »Wie Stars die Krise überstehen«, und zwar unter der Oberüberschrift »Nur die Liebe zählt«. Hier berichten mehr oder weniger Prominente von den Herausforderungen im isolierten Beziehungsalltag,^4^ aber der Artikel steht im Kontext einer durchschnittliche Leser:innen adressierenden Ratgeberspalte, in der Tipps für das Paar-Verhalten in der Krise gegeben werden.^5^ Auch hier geht es um potenzielle oder wirkliche Krisen in der Krise, nämlich Haushalts- und Beziehungsstress in der Pandemie. Zwar wird die pandemische Krise hier nicht, wie in den zuerst genannten Beispielen, en passant mitgeführt, sondern als Ursache der Alltagsprobleme benannt. Im Kern handelt es sich dennoch um alles, was auch ohne Pandemie an Alltagsthemen zu erwarten wäre. Vermutlich liegt darin der Grund, warum sich die Aufmerksamkeit oftmals von dem Virus selbst auf die Seuchenbekämpfungsmaßnahmen verschiebt. Das Virus ist eigentlich Ursache des Ausnahmezustandes. Deren sichtbarer Teil sind aber die Schutzmaßnahmen, die sich auf den Alltag beziehen, und obwohl sie die Lösung darstellen, sehen sie nun aus wie das Problem. Ganz allgemein finden sich zahlreiche Hinweise, wie man mit der Isolation umgehen kann, z.B. »Backen in der Krise« und »Gute Laune trotz Quarantäne«^6^. Außerdem gibt es »Streaming-Tipps für #stayhome«^7^. Indem Ratschläge erteilt werden, wie man mit der häuslichen Situation umgehen kann, werden Maßnahmen gegen die Auswirkungen der Maßnahmen empfohlen, wofür die Zeitschriften ja tatsächlich Expert:innen sind. Dies bezieht sich z.B. auch auf den Umgang mit unterbliebenen Friseur:innenbesuchen. Am 08. April bezeichnet die *inTouch* Carmen Geissens Unfrisiertheit selbst noch als »Luxus-Problemchen. Die Fans sind total genervt«^8^. Der kritische Ton und die Meta-Perspektive sind am 30. April verschwunden, als ein Foto aus der gleichen Strecke noch einmal in der *inTouch* auftaucht. Diese Mal wird das Bild aber gänzlich unironisch als »Beauty-Burnout«^9^ deklariert. Szenarien in Krankenhäusern, Krankheit und Tod wurden vergleichsweise kaum thematisiert. Indem seitens der Zeitschriften kommuniziert werden musste, dass man sich der Krise bewusst ist, ohne hinsichtlich der Themenselektion auf das eigene Programm zu verzichten, kann bei der Lektüre durchaus der Eindruck entstehen, dass ein paar Wochen zu Hause bei der Familie zu verbringen, nur mit Survival Tipps überlebt werden kann, und dass die häusliche Isolation schon zum *worst case* der Pandemieeffekte zählt. Als seien wir in Deutschland überhaupt je eingesperrt gewesen, heißt es dann am 13. Mai in *Closer*: »Hurra, wir dürfen wieder raus«^10^. Dennoch heißt es noch im Juni übertreibend, seit den letzten Monaten »mussten die Staatsoberhäupter drastische Maßnahmen ergreifen. So sind wir alle oder fast alle eingesperrt«^11^. Es ist bezeichnend, dass zwischen März und Mai 2020 sehr häufig pauschal von der negativ konnotierten ›Quarantäne‹ die Rede war – einem Wort, das eine mehr oder weniger medizinische Definition hat und die medizinische Isolation akut kranker oder potentiell kranker Personen meint –, auch wenn eigentlich fast immer einfach nur das *social distancing* gemeint war. – Erst im Zuge höherer Inzidenzen viel später, als sich also die echten Quarantäne-Fälle häuften, wurde Quarantäne wieder als Quarantäne gebraucht. – Die Umsemantisierung dient der Dramatisierung eines vergleichsweise erträglichen Umstands unter Absehung der Gefährlichkeit des Virus. Natürlich entspricht das nicht dem Gesamtbild. Es werden vereinzelt durchaus ernste Töne angeschlagen und Covid-bedingte Todesfälle thematisiert, z.B. von Jörn Kubicki und Roy Horn.^12^ Die – vermutete – Angst der Queen um ihren Mann kam zum Ausdruck: »Geliebter Philip, bitte bleib bei mir«^13^. Und der Mann von Viktoria von Schweden wird als prominenter Risikopatient vorgeführt.^14^ Es kommt also durchaus auch vor, dass vulnerable Gruppen mit prominenten Gesichtern versehen werden. Außerdem wird gelegentlich das – nur potenziell – entspannende oder entschleunigende Moment des Stillstands erwähnt. Dass Eltern einer großen Herausforderung gegenüberstanden, als sie zwischen Home-Office und Home-Schooling rotierten, wurde – bis auf wenige Ausnahmen – nicht fokussiert, sondern sofern kein Lagerkoller und keine Beauty-Probleme zu verzeichnen waren, wurde kommuniziert, dass die Isolation eine Chance bietet: »Faulsein ist ausdrücklich erlaubt«^15^. Im Juni 2020 wurden zahlreiche Seuchenbekämpfungsmaßnamen, z.B. Aspekte des so genannten Lockdowns herunterreguliert. Zu diesem Zeitpunkt berichtet z.B. *inTouch*, dass Carmen und Robert Geiss, auch »die Geissens« genannt, trotz noch bestehender Reisewarnungen einen Trip nach Südtirol unternommen haben.^16^ Es wird Ausbruchsstimmung verkündet. So z.B. auch in folgender Überschrift: »Pleite-Schock auf Mallorca. Ausnahme-Zustand nach der Corona-Krise«^17^. Für die *inTouch *war im Mai 2020 die Corona-Krise offenbar in dem Augenblick beendet, in dem die Maßnahmen endeten, weil die Zeitschrift den Fehlschluss ausdrücklich artikuliert hat, dem auch andere erlegen waren, nämlich, dass man die Lösungsmöglichkeiten mit dem Problem verwechselt, die Präventionsmaßnahmen und nicht das Virus zumindest implizit als Ursache aller Unannehmlichkeiten und Probleme im Blick hatte. Schon zu Beginn der Pandemie war zu verzeichnen, dass in Deutschland oder überhaupt in ›westlichen‹ Ländern bei manchen Personen und Gruppen nur eine irrational geringe Frustrationstoleranz gegenüber Einschnitten in ihre Konsum- und Lifestyle-Gewohnheiten zu verzeichnen sind. Solange die Zeitschriften in Bezug auf Covid bei ihrem ›entdifferenzierten Spezialthema‹ Lifestyle blieben und in hohem Maß als Resonanzraum für Stimmungen fungierten, haben sie möglicherweise diese Befindlichkeiten bestärkt. Abgesehen davon, dass Wirkhypothesen immer heikel sind, gab es auch andere Reaktionen in der Bevölkerung und andere Artikel in den Zeitschriften. Aber auffallend ist eben, dass der ›Kontakt‹ mit der Pandemie seitens der Zeitschriften zunächst einmal den *bottom up*-Prozess initiiert, Menschen medial miteinander zu koppeln, die physisch nun etwas isolierter sind, um eine wechselseitige Versicherung der Aufregung – über die Maßnahmen – vorzunehmen. Auch die anderen verzichten und leiden darunter – so die Kontaktszene Boulevardzeitschriften zu Beginn der Pandemie. Diese Kommunikation wirkt symmetrisch, weil hier aus dem Volk dem Volk Befindlichkeiten vermittelt wurden.

Nun muss unter den Magazinen differenziert werden. Man ist oftmals geneigt, in diesem populären Segment im negativen Sinn des Wortgebrauchs ohnehin ›Oberflächliches‹, ›Sensationalistisches‹ und auch ›Populistisches‹ vorzufinden. Aber in dieser Hinsicht gab es unter den Zeitschriften Unterschiede, die allerdings hier nicht im Einzelnen erörtert werden, weil es dafür einer systematischeren quantitativen Inhaltsanalyse bedürfte. Die *Brigitte* besetzt allerdings ganz klar das seriöse Spektrum unter den Unterhaltungszeitschriften. Es muss aber nicht eine Dichotomie zwischen ›oberflächlich‹ und ›tief‹ aufgemacht werden, um die *Brigitte* zu beschreiben. Sie unterscheidet sich strukturell von den anderen Zeitschriften durch einen anderen zeitlichen Horizont. Insgesamt orientiert sich die Themenauswahl und -setzung weniger an punktuellen Phänomenen. Bei gleicher monatlicher Frequenz wie viele der anderen Zeitschriften artikuliert sie dennoch keine akute Aktualität – sofern man überhaupt in Bezug auf Zeitschriften von einer solchen angesichts von anderen tages- und minutenaktuellen Medien sprechen kann. Allein dadurch ist die Möglichkeit ausgebremst, erste Reflexe auf und Affekte in Bezug auf die Pandemie abzuschöpfen und zu verstärken. Und so thematisiert die *Brigitte *Covid, indem sie herausstellt, welche (Arbeits‑)Leistungen Frauen in der Pandemie leisten. Statt um Konsum geht es um strukturelle Herausforderungen im Alltag, wie Vereinbarkeit von Familie und Beruf, sowie Wohltätigkeit und soziales Engagement von Frauen etc. Auch in anderen Magazinen sah man Prominente, die sich für das Zuhausebleiben bzw. #stayathome ausgesprochen haben. Indem eine Lösungsmöglichkeit vorgeführt wird, verweisen die Zeitschriften auch auf das Problem von Geschäftsinhaber:innen: »Mein Vermieter erlässt mir die Miete«^18^. Es gibt Tipps rund um Urlaubsstornierungen. Video-Telefonie-Anbieter werden getestet. Über ausstehende und aufzuschiebende Routine-Operationen wird informiert. Mai Thi Nguyen-Kim wird als gute Wissenschaftspopularisiererin gefeiert,^19^ und da es diese Zeitschriften sprachlich oft nicht genau nehmen, wird Bill Gates in einer Bildbeschreibung zum »Mann, der Corona-Impfstoff entwickelt«.^20^ In der *Brigitte* vermehrt und dort, wo die übrigen Zeitschriften ihrer – untergeordneten – Informationsfunktion nachkommen, findet eine *top down*-Kommunikation statt, in deren Rahmen die Zeitschriften eine Wissensvermittlungsfläche für Leser:innen bilden. Wo sonst kämen sie niederschwellig in Kontakt zu juristischen Auskünften etc. Dort, wo Video-Telefonie-Anbieter getestet werden und Mai Thi Nguyen-Kim vorgestellt wird, stellen die Zeitschriften eine Kontaktszene im Sinne der Volksbildung und entsprechend erwünschter Verhaltensmodifikationen dar, und in diesen Momenten scheint eben doch die Gefährlichkeit der Pandemie selbst durch. Dies war allerdings, wie bereits gesagt, nicht die dominante Kommunikationsweise in den ersten Monaten.

Unter den untersuchten Zeitschriften gibt es auch solche, die eine stärkere Spezialisierung aufweisen: Mode-Zeitschriften, die allerdings durchaus auch andere Themen beinhalten. In diesen wird weniger versucht, das Populäre mit dem Populären zu würzen, also das Populäre der Prominenten mit dem Populären der Pandemie aufzuladen. Das Thema Covid taucht hier viel seltener auf. In der *inTouch*, die Mode am Rande neben allen anderen Themen verhandelt, muss Mode noch damit verknüpft werden, dass sie zur Pandemie passt. Es gibt Tipps für stylishe Gummistiefel und Regenmäntel, weil man ja noch spazieren gehen dürfe,^21^ was einmal mehr an die Lockdown-Maßnahmen erinnert. Die stärker auf Mode spezialisierten *MySelf, InStyle, Gala Style, Cosmopolitan* und *Vogue* dagegen bringen sehr wenig zu Covid. Die *Vogue* nutzt noch nicht einmal offensichtliche Vorlagen, um ihre Themen an das populärste Thema des Jahres zu knüpfen. In der *Vogue Business* werden Frauen in wichtigen und interessanten Berufen vorgestellt, und hier geht es um Anne Flörcken aus der Berliner Charité, ihres Zeichen Spezialistin für metastasierende Nierenzellenkarzinomen.^22^ Jede andere Zeitschrift hätte vielleicht hektisch nach einer Virologin gesucht. Nicht so die *Vogue Business*, die zwar eine andere Spezialisierung als die *Vogue* aufweist, aber von der *Vogue* herkommend auf distinguierende, ›sophisticated‹ Themen setzt und sich nicht von der Pandemie irritieren lässt. Jedenfalls war dies ganz zu Beginn der Fall, aber da der Vorlauf der Heftplanung nicht bekannt ist, die Artikel aber nicht sehr aktuell sein müssen, ist es gut möglich, dass die Ausgaben schon organisiert waren, bevor die Pandemie an Relevanz gewonnen hat. Denn etwas mehr auf Covid bezogen war die Juni-Ausgabe der *Vogue*. In dieser fällt zwar auch das falsch semantisierte Wort »Quarantäne«^23^, aber die wenigen anderen Bezüge auf Covid sind originell, differenziert und auf Mode und Kunst bezogen. So gibt es einen Artikel zum Verhalten der Modehäuser in der Krise^24^ und einen zur Verlagerung von Kunst und Kultur in die Virtualität: »Die Kultur kämpft und sie blüht im Netz«^25^. Vor allem aber ist das Heft dem Phänomen der Liebe gewidmet, wo wir doch so wenig taktilen und olfaktorischen Kontakt zu unseren Mitmenschen hatten,^26^ was die Entbehrungen der Menschen zu Beginn der Pandemie positiv rahmt und einhegt.^27^

## Phase Oktober bis Dezember 2021

In der winterlichen Jahreszeit Ende 2021 dreht sich vieles um Weihnachten und Silvester. Dementsprechend können sich die Zeitschriften ihren Kernkompetenzen wie Kochen, Kleidung, Schminken, Schenken etc. widmen. In dieser Phase hat sich der Ausnahmezustand bis zu einem gewissen Grad normalisiert, und es haben sich Routinen im Beobachten und Reflektieren der Pandemie eingestellt. Daher kann das Thema durchaus auch einmal ruhen, und es werden Schminktipps für Weihnachten erteilt, ohne dass zwingend auf dessen fragilen Status hingewiesen werden muss. Ab und zu kommt Freude über ein vergleichsweise normales Weihnachtsfest auf: »nach dem Lockdown im letzten Jahr ist es einfach schön, dass wir jetzt viel zuversichtlicher Richtung Weihnachten starten können«.^28^ Über Partys mit Freund:innen heißt es: »wie cool (und normal) ist das denn bitte?«^29^, aber »Natürlich alles ganz coronakonform«^30^. Hin und wieder ist dagegen Wehmut im Spiel, wenn z.B. artikuliert wird, dass man sich um das Fest betrogen fühlt und man »[m]ehr Corona-Sorgen denn je«^31^ hat, oder wenn betont wird, dass Weihnachten *auch* im kleinen Kreis friedlich und liebevoll gefeiert werden kann,^32^ was trotz der positiven Aussicht Abstriche impliziert. Der Ton ist insgesamt ernster geworden. »Nach diesen harten Jahren der Pandemie«^33^ sind diverse Prominente froh, wenn sie einfach ihre Familie wiedersehen können. Aber angesichts dessen, dass nun häufiger die Gefährlichkeit des Virus thematisiert wird, erscheinen Entbehrungen aller Art insgesamt nicht mehr so beklagenswert wie in der ersten Phase. Nicht die Maßnahmen, sondern das Virus ist nun schuld am Ausnahmezustand. »Liebe Leserinnen, liebe Leser, ohne Zweifel, es sind leider immer noch schwierige Zeiten: Das Corona-Virus hat uns fest im Griff.«^34^ Angesichts dessen, wünscht man sich »nur eins: Gesundheit!«^35^ Tony Marshall ist froh, dass er dank seiner Impfung als Risikopatient Covid überlebt hat, und die anderen prominenten Risikopatient:innen bleiben weiterhin vorsichtig.^36^ Dass auch Royals 2021 auf das eine oder andere Fest verzichten mussten, wird eher selbstverständlich ohne Jammerton thematisiert: Prinzessin Amalia aus den Niederlanden durfte ihren 18. Geburtstag nur im kleinen Kreis feiern,^37^ Prinzessin Aiko aus Japan erging es so mit ihrem 20.^38^ Gar kein Bankett in Bayern wegen zu hoher Inzidenz gab es für Margrethe von Dänemark.^39^ Und viele prominente Großeltern, Eltern und Ehepartner:innen waren im letzten Jahr aufgrund von Reisebeschränkungen von Familienmitgliedern getrennt.^40^ Über die Theologin Melanie Wolfers und ihren Ratgeber zu Zuversicht wird mehrfach berichtet und die resignierte Frage in den Raum gestellt: »Nur wie kann das gelingen in diesen Zeiten?«^41^ Im Interview mit der Medizinethikerin Prof. Dr. med. Alena Buyx wird die Frage gestellt, ob die Pandemie unser Verständnis von Ethik geändert hat.^42^ Aber weil Ethik – außer vielleicht als Teilbereich von Philosophie und Theologie – keine Angelegenheit ist, für die es per se Expert:innen gibt, können sich die Magazine auch mit alltagsethischen Erwägungen einschalten, etwa indem konstatiert wird: »Die ich-zentrierten Zeiten sind vorbei, Freiheit ist ohne Verantwortung nichts wert.«^43^ Einer wütenden Krankenschwester wird das Wort erteilt, die über das politische Versagen beim Pandemiemanagement klagt, die darauf hinweist, dass der Freedom Day trotz unterbesetzter Kliniken für den Wahlkampf missbraucht worden sei, und die betont, dass die vierte Welle niemanden hätte überraschen dürfen.^44^

Der ernste Ton geht damit einher, dass auch in Bezug auf Lifestyle-Themen an diesen Stellen eine – eher implizit bleibende – belehrende und wissensvermittelnde Kommunikation stattfindet. Die Kontaktszene Zeitschriften löst sich dort, wo die Zeitschriften auf Covid Bezug nehmen, von ihrem entdifferenzierten Leistungsspektrum, um ausgehend vom virologischen und epidemiologischen Kenntnisstand *top down* für die Gefahren der Pandemie zu sensibilisieren und ein Bewusstsein dafür zu schärfen, dass das Virus selbst den Ausnahmezustand bedingt, auch wenn an den zitierten Stellen nicht mitkommuniziert wird, dass hier eine Vermittlung medizinischen Wissens impliziert ist. Explizit und geradezu didaktisch im Sinne der einhelligen wissenschaftlichen und politischen Einschätzung versuchen die Magazine die Impfung im Allgemeinen und den Booster im Speziellen zu bewerben. »Jetzt boostern, Weihnachten sicher sein«^45^. Während Impfverweigerung als zu bereuender »Leichtsinn«^46^ ausgewiesen wird, findet der Hashtag #AllesindenArm sowie zum Beispiel die Haltung der Schauspielerin Natalie Wörner, nur mit Geimpften zu arbeiten, affirmierend Erwähnung,^47^ und es wird darauf verwiesen, dass viele andere Maßnahmen zurückgefahren werden könnten, wenn wir eine höhere Impfquote hätten.^48^ Informationen zu Impfungen – auch zu neuen Impfstoffen – werden nicht selten explizit von medizinischen Expert:innen bezogen. Der Virologe Dr. Jan Leidel wird allgemein zu Impfungen befragt,^49^ Booster-Empfehlungen der STIKO mitgeteilt^50^ – die gleichwohl kurz nach Erscheinen der Zeitschriften teilweise schon veraltet waren –, Berechnungen der HU Berlin zur geringeren Ansteckungsgefahr durch Geimpfte werden präsentiert,^51^ Prof. Hubert Wirtz von der Post-Covid-Ambulanz in Leipzig klärt über Long Covid auf,^52^ Prof. Gernot Marx erklärt, was es mit Impfdurchbrüchen auf sich hat.^53^ Hier spricht nicht die Prominenz der Pandemie, wie z.B. Christian Drosten und Sandra Ciesek. Das hat aber den Nebeneffekt, dass das Populäre der Pandemie hier weniger zum Ausdruck kommt, insofern als eigens auf den Expert:innenstatus verwiesen wird, indem die Titel, Arbeitsorte und Fachgebiete der Personen einer Vorstellung bedürfen und entsprechend vorgestellt werden. Hier wird auf Professionalisierung statt auf Populäres gesetzt und verdeutlicht, dass auch unbekanntere Personen mehr zu dem Thema zu sagen haben, wenn sie qua Ausbildung dafür spezialisiert sind, als manch bekanntes Gesicht – wobei hier natürlich nicht Drosten und Ciesek gemeint sind, bei denen das eine das andere nicht ausschließt. Wo die Expert:innen zu Wort kommen, wird auch auf die interne Differenzierung und Spezialisierung der Zeitschriften gesetzt, d.h. zumeist befinden sich die Informationen in den jeweiligen Gesundheitsratgeberressorts der Magazine.

Mindestens *Frau im Spiegel, neue woche* und *tina* scheinen in ihren Impfkampagnen mit ihrer Wortwahl auf die Feigheit oder Kindlichkeit von Impfverweigernden anzuspielen, indem die Impfung als »Piks« bezeichnet wird.^54^*Die Neue Post* unterstellt direkter eine Angststörung, wenn sie darauf verweist: »Gegen Spritzen-Phobie kann man etwas tun.«^55^ Man kann sich des Eindrucks nicht erwehren, als sei diese kleine Spitze ein rhetorischer Trick, um Impfgegener:innen herauszufordern. Gefühltes rebellisches Held:innentum wird hier nicht in seiner politischen Dimension betrachtet – und auf diese Weise ernst genommen –, sondern der Lächerlichkeit preisgegeben, indem vordergründig Ängste ernst genommen und entkräftet werden. Falls es Impfgegner:innen wirklich um Ängste und Zweifel ginge – so suggeriert es die Wortwahl –, ranken diese sich doch nur um eine Kleinigkeit, und vor allem können diese schließlich überwunden werden. In dieser Logik wird deutlich, dass unüberwindbare Impfängste vermutlich eben keine Impfängste sind, sondern politisches Kalkül spezieller Gruppierungen. Auch *Frau im Spiegel* diskreditiert Impfgegner:innen nicht direkt, sondern nimmt deren Argumente zunächst einmal ernst oder unterstellt einen Rest an Rationalität, wenn sie die ›klassisch‹ hergestellten Impfstoffe in Anschlag bringt: »Berechtigte Hoffnung, dass sich Impfzögerer doch noch ihren Piks abholen, machen Vakzine, die nach klassischen Verfahren hergestellt werden«^56^. Insgesamt findet in dem Segment eine klare Positionierung pro Impfung statt; es gibt kleine Seitenhiebe, aber man ist bemüht, Impfgegner:innen nicht durch allzu harte Kritik von vorne herein zu verprellen. In jedem Fall schöpfen die Zeitschriften hier ihre Expertise in Sachen positive Leser:innenansprache aus. Und dabei wird die Balance gewahrt zwischen tantenhafter, freundschaftlicher Ansprache auf Augenhöhe und dem Zitieren medizinischer Expert:innen. Insgesamt taucht das Thema Covid auch unabhängig von Impfungen nun weit häufiger in Bezug auf medizinische Fragen auf als in der ersten Phase, was daran liegt, dass sich ein gewisser Wissensstand konsolidiert hat, der nun auch im Wochen- und Monatsrhythmus kommuniziert werden kann. Dabei reicht das Spektrum vom individuellen wie gesetzlich verordneten Schutz vor der oder Erkennen/Testen der Krankheit selbst^57^ über Mutationen^58^ – wobei Omikron die Zeitschriften schnell veraltet wirken lässt – zu den unerwünschten Nebenwirkungen der Maßnahmen wie Depression durch Isolation^59^. Wie bereits eingangs erwähnt, befinden sich unter den Expert:innen für Pandemie nicht nur Mediziner:innen und Naturwissenschaftler:innen, sondern die Pandemie hat schließlich auch eine rechtliche Seite. Dem werden die Zeitschriften in entsprechenden Ratgeber-Spalten gerecht, in denen Jurist:innen arbeits^60^- und steuerrechtliche^61^ Fragen erörtern, Reiserückerstattungsmodalitäten diskutieren^62^ und mögliche Verstöße gegen Corona-Auflagen behandeln.^63^

Insgesamt ist zu beobachten, dass die Pandemie in Lauf der Zeit doch eine disruptive Kraft hatte und dass in den Zeitschriften eine Refiguration hin zu Wissens- und Wissenschaftsvermittlung stattgefunden hat, das heißt auf lange Sicht haben sich die Zeitschriften im Großen und Ganzen von der Pandemie irritieren lassen, ihre phatisch-populäre Kommunikation zurückgefahren, in der sich Lai:innen in ihrer Befindlichkeiten wechselseitig bestärken konnten, und eine kontaktstiftende Funktion zwischen Wissenschaft und dem Lai:innenpublikum erfüllt, und das für eine Altersgruppe, die zum einen vergleichsweise stark betroffen ist und zum anderen seltener an anderen sozialen Medien partizipiert. Nicht nur sind die Zeitschriften insgesamt bemüht, Expert:innen sprechen zu lassen, sondern sie thematisieren auch die Pseudo-Expertisen auf dem Gebiet. So heißt es über Michael Wendler, er verbreite Fake News zum Tod von Mirco Nontschew, indem er diesen mit Impfungen in Verbindung bringt, was »leider« keine rechtlichen Konsequenzen für Wendler habe.^64^ An anderer Stelle wird Wendler als »Verschwörungsschwurbler« bezeichnet.^65^ Da die Magazine an ernsten Konfliktthemen wenig Interesse haben, geraten sie nicht in Versuchung, durch *false balancing* die Stimme der Covid-Leugner:innen und Impfgegner:innen überzubewerten. Sie berichten über Verschwörungsnarrative, Impfgegner:innen und Querdenker:innen ziemlich selten, aber sie blenden das Thema nicht aus, was zu einer ausgewogenen Berichterstattung dazu gehört. So wird Vicky Krieps, die sich an der #allesdichtmachen-Aktion beteiligt hatte, völlig neutral in ihrer Haltung präsentiert, dass sie schließlich finanzielle Probleme in der Krise hatte und als Künstlerin nicht immer politisch korrekt sein müsse. – Bemerkenswert ist, dass sich laut Krieps einzig Daniel Kehlmann nach der Aktion bei ihr gemeldet hatte und wohl ebenfalls der Meinung ist, man würde ja wohl noch was sagen dürfen...^66^ – Das komplettiert das Bild des Coviddiskurses und es werden Gegensätze jeweils in einem Medium in Kontakt gebracht, ohne dass sich die Zeitschriften zu stark an den problematischen Positionen abarbeiten und sie damit ungewollt in Szene setzen.

Es würde den vorliegenden Rahmen sprengen und Redundanzen erzeugen, das gesamte, sehr breit gewordene Themenspektrum rund um Covid aufzuzählen. Die Breite resultiert nun aber nicht daraus, dass jedes übliche Thema assoziativ damit aufgeladen wird, dass es nun eben *auch* unter Pandemie-Bedingungen nach wie vor ein Thema ist, wie dies in der ersten Phase oft der Fall war. Vielmehr sind nach eineinhalb Jahren mehr als zuvor alle Lebensbereiche kausal von der Pandemie betroffen. Stricken kann man nun nicht *auch* in der Pandemie, sondern die Pandemie hat es aufgezwungen, häusliche Hobbys zu entdecken.^67^ Kochrezepte sind nicht *auch* in der Pandemie nach wie vor hilfreich, sondern die Pandemie hat aufgrund geschlossener Restaurants ein angestrengtes Verhältnis zum Kochen hervorgerufen.^68^ Abgesehen davon wird thematisiert, dass häusliche Gewalt, Überforderung, Langeweile, Nähe- und/oder Abgrenzungsbedürfnisse nicht allgemein interessante Themen sind, sondern durch Covid eine neue Dimension erhalten haben.^69^

## Überlegungen

Printmedien haben in den letzten Jahren aufgrund von Netzangeboten deutlich an Reichweite verloren. Aber gedruckte Unterhaltungsmagazine sind für Leser:innen als Bündel aus Ansprache – zum Beispiel im Editorial –, Unterhaltung und Beratung, Kreuzworträtsel und Gewinnspiele sowie Leser:inneneinsendungen nach wie vor beliebt. Sie sprechen die Leser:innen mit je eigenem Ton an und vermögen, sie an sich zu binden. Laut Statista.com hat die *Freizeit Revue* im ersten Quartal 2022 eine Auflage von 487.052 Exemplaren; *Frau im Spiegel* 164.261; viele der anderen gelesenen Blätter bewegen sich in der Mitte.^70^ Auch wenn man daraus nicht schließen kann, wie viele Menschen tatsächlich wie regelmäßig Unterhaltungsmagazine lesen, handelt es sich bei den Magazinen um ein im Vergleich mit Online-Angeboten, sozialen Medien und Messenger-Diensten unterschätztes Medium, wenn es um die Frage geht, wie Wissen popularisiert wird oder Diskurse im Populären verhandelt werden. Die Zeitschriftenforschung hat in den letzten Jahren zu Recht einen bedeutenden Aufschwung erfahren.^71^ Zeitschriften nun als Kontaktszenen zu begreifen, bedeutet zunächst einmal, in einem vielleicht etwas tautologischen Sinne sie als Teil eines kommunikativen Kontaktes zwischen sich und ihrer Leser:innenschaft zu betrachten. Vielmehr sind die Zeitschriften aber als Ganze selbst je spezifische multimodale und interdiskursive Kontaktszenen, in denen entweder aktuelle Diskurse gesammelt und nebeneinandergestellt werden oder aber vermittelnd in Bezug gesetzt werden können. Wie dynamisch sich Kontaktszenen verhalten, lässt sich daran beobachten, dass sich die Zeitschriften letztlich durch die Pandemie haben irritieren lassen und ihr Programm refiguriert haben.

In der ersten Screening-Phase haben die meisten Magazine zunächst als Sammelsurien für die bekannten unterhaltenden Themen fungiert, in denen die Pandemie *irgendwie auch* thematisiert wurde, wodurch sich nicht nur, aber in großem Maß *bottom up* Lifestyle-Befindlichkeiten artikuliert wurden. Das heißt, es stellte sich zuerst eine selbstreferentielle Schleife quasi-phatischer Kommunikation ein, in der überreaktiv jedes Alltagsphänomen, jedes Problem, jede Krise nun an Covid geknüpft wird, um zu zeigen, dass man weiß, woran Leser:innen denken. Zeitschriften müssen mitklatschen und -tratschen, das heißt sie müssen mindestens monatsaktuell zeigen, dass sie auf der Höhe der Zeit sind, und weil sie dabei auch unterhaltsam sein müssen, setzten sie zu Beginn den Akzent auf ihre Kernkompetenz Lifestyle oder verhinderter Lifestyle. Dabei schöpfen sie die ersten – zu einem größeren Teil dysfunktionalen – Reflexe auf die Pandemie ab und fungieren möglicherweise als deren Verstärker. Ausnahmen bildeten die Zeitschriften, die einen stärkeren Grad an Spezialisierung aufweisen, wie Modezeitschriften, und die *Brigitte*. In der zweiten Screening-Phase zeigt sich eine stärkere Spezialisierung und Differenzierung. Covid-bezogene Kommunikation wurde deutlicher von den Lifestyle-Themen getrennt in einschlägigen Ressorts verhandelt und an Expert:innen abgegeben. Hier findet *top down* direkte Wissens- und Wissenschaftsvermittlung statt. Strukturell vollzieht sich hier ein Übergang vom Freund:innenschafts- zum Expert:innenmodus, auch wenn der freundschaftliche Ton in den redaktionellen Teilen nicht aufgegeben wird. Diese Asymmetrisierung von Expert:innen und Lai:innen gewährt Leser:innen den Kontakt zu einem entfernt operierenden System, nämlich der Wissenschaft, obwohl Unterhaltungszeitschriften dafür per se nicht zuständig sind. Da in der Zwischenzeit – wiederum in den Massenmedien – sehr viel über das Verhältnis von Lai:innenempfindungen und Expert:innenwissen debattiert wurde, ist nicht auszuschließen, dass hier ein bewusster Lernprozess stattgefunden hat. Fragt man sich nun, warum dies zu Beginn anders war, so ist festzuhalten, dass Unterhaltungsmagazine nicht ohne Routinen auskommen und sie sich nur schwer in aktuelle Themen einfinden, gleichzeitig aber halbwegs aktuell sein müssen. Sie können erst dann Expert:innenwissen popularisieren, wenn dieses konsolidiert ist. Dann greift auch die interne Differenzierung der Blätter, und die Gesundheitsratgeber können spezifisch ausgenutzt werden. Solange dies nicht seriös möglich war, man aber zum Thema nicht schweigen konnte, musste wohl auf diffuse und unspezifische Weise die Popularität der Pandemie selbst zum Thema gemacht werden. Die Zeitschriften können ein eigenes – diffuses – Skript liefern, wenn es darum geht, aktuelle Stimmungsbilder zu reflektieren oder zu erzeugen; sie setzen aber seriös auf das Skript der Expert:innen, sobald dieses ansatzweise zur Verfügung steht. Insofern ist hier eine deutlich geringere selbstreferentielle Dynamik populärer Empfindungen zu verzeichnen als in manchen (!) Blasen sozialer Medien, die sich nicht durch neue Sachstände und Informationen irritieren lassen. Obwohl die Pandemie-Krise für die Zeitschriften insofern einen disruptiven Effekt hatte, als besagte deutliche Umstellung des Umgangs mit der Pandemie zu verzeichnen ist, lesen sich die einzelnen Ausgaben dennoch nicht als Resonanzkörper für den Ausnahmezustand. Solange die Krise an Beautykatastrophen, Beziehungsprobleme und Langeweilebewältigungsmaßnahmen gekoppelt ist, fällt sie nicht eigens auf, und dort, wo medizinisches Fachwissen eine Rolle spielt, ist der Ton sehr sachlich und die Handhabung lösungsorientiert.

## Footnotes

1 Vgl. die reichhaltige Nachrichtenwerttheorie-Forschung, die 1922 mit Walter Lippmann ihren Anfang hatte, oder im Wirkbereich, die Agenda-Setting-Forschung seit den 1960er-Jahren, die mit Bernhard Cohen, Maxwell McCombs und Donald Shaw verbunden ist.

2 *Closer*. 06. Mai, S. 13.

3 *Brigitte*. 20. Mai, S. 82.

4 *Gala*. 9. Apr., S. 20.

5 *Gala. *9. Apr., S. 24.

6 *Frau im Spiegel*. 15. Apr., S. 11.

7 *inTouch*. 30. Apr., Cover.

8 *inTouch*. 08. Apr. S. 7.

9 *inTouch*. 30. Apr. S. 7.

10 *Closer*. 13. Mai, S. 4.

11 *What’s up*. Nr. 20, Jun., S. 23.

12 *Neue Post*. 6. Mai, S. 6; *Das Goldene Blatt*. 18. Mai, S. 70 u.v.m.

13 *Neue Post*. 6. Mai, Cover.

14 *Frau im Spiegel*. 01. April, S. 12.

15 *inTouch*. 30. Apr, 16.

16 *inTouch*. 18. Jun., S. 14.

17 *inTouch*. 20. Mai, Cover.

18 *Neue Post*. 06. Mai, S. 26.

19 *Gala*. 7. Mai, S. 68.

20 *Bunte.* 7. Mai S. 18.

21 *inTouch.* April-Ausgabe, S. 23.

22 *Vogue.* April-Ausgabe, S. 47.

23 *Vogue*. Juni-Ausgabe, S. 18.

24 *Vogue*. Juni-Ausgabe, S. 38.

25 *Vogue*. Juni-Ausgabe, S. 50.

26 *Vogue*. Juni-Ausgabe, Editorial.

27 Im Kontext der zweiten Phase wird auf *Brigitte* und die spezialisierten Modezeitschriften kaum noch Bezug genommen, weil sich hier keine grundlegenden Änderungen zur ersten Phase zeigen.

28 *Jolie*. Dezember-Ausgabe, Editorial.

29 Ebd.

30 Ebd.

31 *Bild der Frau*. 17. Dez., Editorial.

32 *Gala*. 16. Dez., Editorial.

33 *neue woche*. 17. Dez., S. 18.

34 *Das Goldene Blatt*. 11. Dez., Editorial.

35 *Neue Post*. 15. Dez., Editorial.

36 *Das Neue Blatt*. 15. Dez., S. 5, 74.

37 *Bunte*. 16. Dez., S. 22; Neue Post. 15. Dez., S. 6.

38 *Frau im Spiegel*. 15. Dez., S. 10.

39 *Frau im Spiegel*. 24. Nov., S. 26. Royals *never complain*, weshalb diese in jeder Phase die Fassung bewahrten bzw. so präsentiert wurden, das heißt die Thematisierung dieser Gruppe muss von der der anderen Prominenten unterschieden werden, die in der ersten Phase, wie zuvor beschrieben, durchaus jammern durften (wenngleich dies nicht von allen wurde) und die in der zweiten Phase vermehrt mit ernsterem Habitus präsentiert wurden.

40 *Das Neue Blatt*. 15. Dez., S. 11; Bild der Frau. 17. Dez., S. 27; Frau im Spiegel. 15. Dez., S. 30.

41 *tina*. 15. Dez., S. 6; *Für Sie*. 24. Nov., S. 34.

42 *Elle*. Januar-Ausgabe (erscheint zuvor im Dez. 21), S. 67.

43 *Bild der Frau*. 17. Dez., Editorial.

44 *Bild der Frau*. 5. Nov., S. 5.

45 *tina*. 15. Dez., 50.

46 *neue woche*. 26. Nov., S. 64.

47 *Frau im Spiegel*. 24. Nov., S. 45, 25.

48 *Freizeit Revue*. 3. Nov., S. 74. Vor Weihnachten befinden wir uns noch in der Prä-Omicron-Welle, die in dieser Hinsicht ein Game-Changer war.

49 *Freundin*. 17. Nov., S. 106.

50 *tina*. 3. Nov., S. 55.

51 *tina*. 15. Dez., S. 50.

52 *tina*. 15. Dez., S. 50.

53 *tina*. 3. Nov., S. 55.

54 *Frau im Spiegel*. 15. Dez., S. 25; *tina*. 15. Dez., S. 50; *neue woche*. 17. Dez., S. 62.

55 *Neue Post*. 24. Nov., S. 58.

56 *Frau im Spiegel*. 15. Dez., S. 52.

57 *neue woche*. 17. Dez., S. 49; *Das Goldene Blatt*. 11. Dez., S. 23; *Freizeit Revue*. 24. Nov., S. 71; *Das Neue Blatt*. 24. Nov., S. 54; *Bunte*. 30. Sep., S. 85; *tina*. 3. Nov., S. 56.

58 *Frau im Spiegel*. 24 Nov., S. 52; *tina*. 3. Nov., S. 57.

59 *Für Sie*. 29. Sep., S. 44.

60 *neue woche*. 17. Dez., S. 58.

61 *tina*. 15. Dez., S. 64.

62 *Bild der Frau*. 5. Nov., S. 75.

63 *Freizeit Revue*. 24. Nov., S. 22.

64 *inTouch*. 15. Dez., S. 14.

65 *Closer*. 15. Dez., S. 20.

66 *Bunte*. 4. Nov., S. 88.

67 *Jolie*. Dezember-Ausgabe, S. 11.

68 *Freundin*. 22. Sep., S. 64, 98.

69 *Bild der Frau*. 5. Nov., S. 30; *Brigitte*. 27. Okt., S. 174.

70 IVW (2022).

71 Man denke an den Arbeitskreis Zeitschriftenforschung am ZfL in Berlin, das Teilprojekt zur historischen Zeitschriftenforschung der Verfasserin im SFB 1472 oder an neuere sehr verschiedenartig gelagerte Publikationen wie Pfurtscheller (2017); Stockinger (2018).
